# Chloroform Again

**Published:** 1870-09

**Authors:** E. C. Hamilton

**Affiliations:** Washington C. H., Fayette Co., Ohio


### Editorial.

### CHLOROFORM AGAIN.

By the following it will be seen that another victim to the indiscriminate use of chloroform has fallen.

So great is the danger that attends the use of chloroform as a general anæsthetic in minor surgical operations, and especially in Dental practice, and so often attended with fatal results, that we have for years advised against its use, and have refused to use it or permit it to be used in our practice, for the production of anæsthesia in the extraction of teeth. That death will not occur when proper care is exercised, is simply not true—either this or a great many Dentists and physicians are not capable of exercising proper care.

We have several objections to any general anæsthetic in every day Dental practice, which we may take occasion to speak of ere long. T.

WASHINGTON C. H., FAYETTE CO., OHIO,
Oct. 5, 1870.

DEAR DOCTOR: Another victim to chloroform paid the penalty in my office on Thursday last.

Mrs. Garis, the wife of our county sheriff, came to my office on Thursday afternoon for the purpose of having three roots and one molar tooth extracted. Dr. I. G. Wilson, a physician of this place, was with her, to administer chloroform. After I examined the mouth and laid out the necessary forceps for the operation, Dr. Wilson proceeded with the chloroform, and after administering probably ten minutes (he did not intend to put her entirely under its influence), he called to me to take out one tooth. I then extracted the root of the right superior lateral incisor, only about one-fourth of an inch in length, and attended with no difficulty whatever. The patient then asked for more chloroform, and the Doctor was on the point of giving it, when death ensued. Three physicians worked probably three hours to restore life, but all in vain.

These are the facts in the case. Three of our medical gentlemen, who use chloroform extensively, attempted to charge upon "the shock of extraction" as the cause of death, but the people so ridiculed the idea that they abandoned it. Of course all who are acquainted with the facts attach no blame or responsibility upon me, as it is well known that I have invariably refused to use chloroform, and have advised in every case against its use in Dental surgery.

I make this statement to you so that you may know what the facts are, and not be misled by newspaper statements, in case you desired to refer to the case in the DENTAL REGISTER.

Very truly yours,
E. C. HAMILTON.

